# Comparative in vivo toxicokinetics of silver powder, nanosilver and soluble silver compounds after oral administration to rats

**DOI:** 10.1007/s00204-023-03511-6

**Published:** 2023-05-17

**Authors:** Jelle Mertens, Anissa Alami, Katrien Arijs

**Affiliations:** 1 European Precious Metals Federation, Avenue de Tervueren 168 Box 6, 1150 Brussels, Belgium; 2 ARCHE Consulting, Liefkensstraat 35D, 9032 Wondelgem, Ghent, Belgium

**Keywords:** Silver, Toxicokinetics, Tissue distribution, Nanoparticles, Bioavailability, Copper

## Abstract

Silver (Ag; massive, powder and nanoform) and Ag compounds are used in industrial, medical and consumer applications, with potential for human exposure. Uncertainties exist about their comparative mammalian toxicokinetic (‘TK’) profiles, including their relative oral route bioavailability, especially for Ag massive and powder forms. This knowledge gap impedes concluding on the grouping of Ag and Ag compounds for hazard assessment purposes. Therefore, an in vivo TK study was performed in a rat model. Sprague–Dawley rats were exposed via oral gavage for up to 28 days to silver acetate (AgAc; 5, 55, 175 mg/kg(bw)/d), silver nitrate (AgNO_3_; 5, 55, 125 mg/kg(bw)/d), nanosilver (AgNP; 15 nm diameter; 3.6, 36, 360 mg/kg(bw)/d) or silver powder (AgMP; 0.35 µm diameter; 36, 180, 1000 mg/kg(bw)/d). Total Ag concentrations were determined in blood and tissues to provide data on comparative systemic exposure to Ag and differentials in achieved tissue Ag levels. AgAc and AgNO_3_ were the most bioavailable forms with comparable and linear TK profiles (achieved systemic exposures and tissue concentrations). AgMP administration led to systemic exposures of about an order of magnitude less, with tissue Ag concentrations 2–3 orders of magnitude lower and demonstrating non-linear kinetics. The apparent oral bioavailability of AgNP was intermediate between AgAc/AgNO_3_ and AgMP. For all test items, highest tissue Ag concentrations were in the gastrointestinal tract and reticuloendothelial organs, whereas brain and testis were minor sites of distribution. It was concluded that the oral bioavailability of AgMP was very limited. These findings provide hazard assessment context for various Ag test items and support the prediction that Ag in massive and powder forms exhibit low toxicity potential.

**Supplementary Information:**

The online version contains supplementary material available at 10.1007/s00204-023-03511-6.

## Introduction

Silver (Ag) is a precious metal with a unique combination of properties such as highest electrical and thermal conductivity and lowest contact resistance of all metals, and is utilised for a broad range of industrial, consumer and medical applications. Main uses are in electronics and batteries, solders, jewellery, table- and silverware, dental materials and increasingly as an antimicrobial agent, e.g. within medical devices, biocides or personal care products (Silver Institute 2022). Silver in commerce comprises of simple silver compounds like silver nitrate (AgNO_3_) or silver acetate (AgAc), nanosilver (AgNP) and so-called ‘bulk’ Ag forms such as Ag powder (AgMP) and Ag massive (Nowack et al. 2011; Hedberg and Nordberg 2021). Hence, a potential for human exposure exists for Ag compounds and AgNP as well as for these ‘bulk’ Ag forms, although much recent interest has centred on engineered AgNP.

(Sub)chronic exposure to elevated Ag concentrations might lead to a grey–blue coloration of tissues. This coloration is known as ‘argyria’. Argyria can appear localised or generalised, is related to the deposition of Ag as Ag^0^, silver selenide and/or silver sulphide and is a detoxification mechanism towards silver (due to the low solubility of the Ag deposits) and thus harmless, unless appearing in ocular tissue (cfr. review articles of Hadrup et al. (2014), Hadrup et al. (2018) and Mota and Dinis-Oliveira (2021)). Tissue coloration has been observed with humans (cfr. references in Hadrup et al. 2014 and 2018) as well as with mammals (e.g. Loeschner et al. 2011; Boudreau et al. 2016; Sprando et al. 2017). Elevated systemic silver concentrations are, however, also associated with a possible effect on reproduction and development; although no reproductive toxicity effects have ever been reported for silver massive or powder and several (sub)chronic exposure studies to soluble Ag compounds or AgNP did not show adverse effects in reproductive organs (Kim et al. 2008; Hadrup et al. 2012; Williams et al. 2015) or to reproductive parameters (NTP 2002), some studies did conclude on adverse effects on the developing embryo (Shavlovski et al. 1995; Sprando et al. 2017; Renaut 2022). This scattered database complicates the hazard and risk assessment of silver compounds and—mainly—AgNP, AgMP and massive Ag.

In terms of Ag absorption and distribution phases following intake by mammals, the behaviour of particulate and dissolved Ag species in physiological milieu is complex and dependent on multi-factorial influences. Under physiological conditions, an array of chemical, physical and biochemical processes determines the absorption behaviour of Ag, including complex oxidative dissolution of Ag particles (like AgNP; Liu et al. 2012; Batchelor-McAuley et al. 2014; Molleman and Hiemstra 2017), re-speciation to insoluble (AgCl) or soluble (AgCl_x_^(x−1)−^ with *x* = 2–4) chloride complexes in the gut (Walczak et al. 2013; Kaiser et al. 2017), interaction with or binding to food components or biological thiol-containing molecules (Liu et al. 2012; Behra et al. 2013), AgNP opsonisation with proteins (protein corona formation; Walczak et al. 2013; Durán et al. 2015), interchange of Ag^+^ to secondary AgNP formed in situ (Walczak et al. 2013; Bachler et al. 2013; Juling et al. 2016) and active uptake of Ag^+^ from the gastrointestinal (GI) tract via metal transporter systems (Behra et al. 2013 and citations therein). Hence, unlike some other metals, the application of simplified *in chemico* or in vitro models to evaluate bioaccessibility (as proxy for bioavailability) is not a viable approach for Ag. Instead, there is a reliance on information from in vivo toxicokinetic (‘TK’) investigations.

Whilst a mammalian TK dataset exists for various silver compounds and AgNP, it remains rather fragmentary, especially concerning robust comparative evaluations between the soluble Ag compounds, AgNP and the ‘bulk’ forms (like AgMP). Studies have predominantly been performed in rodent models via the oral exposure route. With respect to derived bioavailability (*F* values) or other estimates of the orally absorbed fraction of the administered dose, Ag is absorbed to a relatively limited extent from the GI tract and approaching 5% as a maximum observed value (Boudreau 2012; Boudreau et al. 2016; Bachler et al. 2013). The water solubility of Ag compounds is not a simple correlate with their relative bioavailability, e.g. the greater water solubility of silver nitrate compared to silver acetate (i.e. 216 g and 1.05 g per 100 mL, respectively) is predicted to not be associated with a distinct absorption profile between the two compounds (Bachler et al. 2013). As another example, silver chloride has very low water solubility, but it has the potential to re-speciate when exposed to gastric free chloride concentrations to absorbable chloride complexes (Levard et al. 2013).

Most in vivo TK studies performed on AgNP of various sizes and capping (coating) systems support a conclusion that AgNP exhibit lower bioavailability than soluble Ag compounds (Bachler et al. 2013; Boudreau et al. 2016; Park 2013; van der Zande et al. 2012). Within this dataset, highest oral bioavailability has typically been found for smaller AgNP in single-dose experiments (Boudreau 2012; Park et al. 2011), i.e. *F* values in the range of approximately 1–4%. However, a confounding consideration is that AgNP formulations contain a co-existing soluble Ag^+^ fraction that might be substantial and even up to 40% or more of the total Ag content (Loeschner et al. 2011; Kittler et al. 2010; van der Zande et al. 2012).

Achieved tissue concentration datasets provide further evidence that soluble Ag compounds have relatively greater systemic availability than AgNP after either single-dose administration (Park 2013) or repeated dosing (Loeschner et al. 2011; van der Zande et al. 2012). Irrespective of route of administration, and whether the administered form was a soluble Ag compound or AgNP, a very common observation in rodent and non-rodent species has been the preferential distribution of Ag to the reticuloendothelial system, mainly to the liver and spleen (Klaassen 1979; Loeschner et al. 2011; Boudreau et al. 2016; van der Zande et al. 2012; Gan et al. 2020). Controversies about the potential neurotoxicity (e.g. Rungby and Danscher 1983a,b; Loeschner et al. 2011; Lankveld et al. 2010) and reproductive toxicity (e.g. Shavlovski et al. 1995; Sprando et al. 2017; Renaut 2022) of Ag have recently focussed attention on Ag levels present in the brain and reproductive tissues. Most TK investigations have shown the brain and testis to be minor sites of distribution, although contradictory reports in rodents after oral administration do exist (Kim et al. 2008; van der Zande et al. 2012). Some studies report accumulation and cytological changes in ovary after repeated exposure to AgNP or AgNO_2_ (Hadek 1966; Song et al. 2013), but reliable data on the extent of Ag distribution to the ovary have so far been lacking. Our study was designed to address these uncertainties and data gaps.

All the aforementioned datasets cover only soluble Ag compounds and nanosilver (the latter mainly comprising AgNP of sizes from 5 nm to ~ 100 nm of either capped or uncapped types). We recognised that no equivalent information existed for ‘bulk’ Ag, such as (fine) powders in the micrometre-size range, even though industrial use of such forms is extensive, with high tonnages being globally marketed (Silver Institute 2022). Hazard assessment of these ‘bulk’ Ag forms has so far been based on read-across of TK and toxicity data from soluble Ag compounds or AgNP, even though a data-driven justification for this approach has been absent. Therefore, the aim of this investigation was to use an in vivo rat model to establish the comparative TK of several Ag test items, i.e. silver nitrate and silver acetate (as soluble Ag compounds), a 15 nm Ag nanoparticle formulation (equivalent to a well-characterised certified reference nanomaterial; EC JRC 2011) and a fine silver powder (0.35 µm particle size) as a conservative representative of ‘bulk’ silver. The oral route study was designed to provide data on comparative systemic exposure to Ag and on differentials in achieved tissue levels. The study was conducted according to Good Laboratory Practice (GLP) principles and in conformance with OECD Test Guideline 417 (OECD 2010). Some previous Ag TK investigations have suffered from methodological deficiencies impacting on their reliability such as divergence from OECD TG 417 norms, inadequate physico-chemical characterisation of the test items (especially as formulated in dosing vehicles), use of inappropriately high-dose levels likely to cause disturbance of kinetics due to excessive toxicity, inappropriate dosing vehicles liable to cause re-speciation of Ag test items, application of blood and tissue Ag extraction techniques which were not optimised for full recovery of Ag, or use of analytical methods with sub-optimal sensitivity (e.g. Kim et al. 2008; Park 2013). Our experimental approach took such considerations into account. Also, it is well established that a key mechanism involved in the systemic toxicity of bioavailable forms of Ag is the induction of an indirect copper deficiency state (Shavlovski et al. 1995; Lison et al. 2021). In ancillary studies, the effects of treatment with either a soluble Ag compounds or Ag powder on circulating copper levels were examined.

## Materials and methods

### Test items

The test items were selected as representative of different classes of either soluble Ag compounds or elemental Ag forms, also with due regard to their established common use in prior TK or toxicity studies:

(i) Soluble Ag compounds: Silver(I) acetate (‘AgAc’; purity > 99.99%) was obtained from Heraeus Deutschland GmbH & Co. KG. and silver(I) nitrate (‘AgNO_3_’; purity 99.97%) was sourced from Ames Goldsmith UK Limited.
(ii) Nanosilver (AgNP): agpure^®^ W10, a small-size AgNP (d50 of 15 nm), equivalent to an OECD and BAM certified reference nanomaterial known as NM-300 (EC JRC 2011), was obtained from RAS AG, Germany. The material is supplied as a yellow–brown aqueous dispersion of nanosilver stabilised in 4% (w/w) polyoxyethylene glycerol trioleate and 4% (w/w) polyoxyethylene (20) sorbitan mono-laurate [Tween 20] (EC JRC 2011).
(iii) Ag powder (AgMP): a fine silver powder of highly uniform spheroidal shape (purity > 99%; d50 of 0.35 µm) was sourced from Ames Advanced Materials Corp., US.

Test item characteristics, including selected physico-chemical characteristics, are provided in Table 1. To fully establish the characteristics of the AgMP and AgNP forms after their suspension in the dosing vehicles, ancillary physico-chemical studies were performed including dynamic light scattering (DLS), microscopy via Scanning Electron Microscopy/Scanning Transmission Electron Microscopy (‘SEM/STEM’), and analysis of ionic silver (Ag^+^) fraction (using 3 kDa filtration followed by inductively coupled plasma optical emission spectroscopy analysis (‘ICP-OES’))—see also Table 1. Further details are provided under Supplementary information.

**Table 1:** Selected test item characteristics

	Silver powder (AgMP)	Nanosilver (AgNP)^a^	Silver(I) acetate (AgAc)	Silver(I) nitrate (AgNO_3_)
Composition/format	Uncoated Ag particulate (crystalline, of highly uniform spheroidal shape)	AgNP in aqueous dispersion; 10.0 ± 0.50 wt% Ag (100 mg/mL); pH 7.0–9.0	Crystalline powder	Crystalline powder
Molar mass	107.87	107.87	166.91	169.87
Particle size characteristics (as supplied)	d90 0.53 μm d50 0.35 μm d10 0.24 μm	d99 20 nm d50 15 nm		
Density	2.34 m^2^/g (by BET^b^)	2.24 m^2^/g (by BET^b^)		
Behaviour in dosing vehicles^c^	Acceptably dispersed suspensions (without evidence of significant sedimentation or agglomeration of particles) following the sonication and vortex mixing procedure		Soluble up to the highest concentration of test item employed. See also Table 2	
Measured ionic Ag fraction (when formulated in dosing vehicles)^d^	0.0004% (MC) 0.001% (5% gluc)	4.8% (MC) 2.5% (5% gluc)		

^**a**^Physico-chemical properties of the equivalent NM-300 Reference Nanomaterial (in native form) have previously been extensively characterised (EC JRC 2011), including: morphology, size distribution, zeta potential, agglomeration behaviour, inherent stability and homogeneity, and silver-ion release characteristic

^b^Brunauer–Emmett–Teller

^c^Oral route: 1% w/v aqueous methyl cellulose (‘MC’) for AgMP and AgNP, or purified water for AgAc and AgNO_3_. Intravenous (‘i.v.’) route: 5% w/v aqueous glucose (‘5% gluc’) for AgMP and AgNP, or purified water for AgAc and AgNO_3_

^d^Values are maximum ionic Ag (Ag^+^) fractions measured after 11 days (formulated in 1% MC), or after 1 day (in 5% gluc)

### Animals

CD Sprague–Dawley (SD) rats of both sexes (Crl:CD IGS strain; specific pathogen-free; 8–10 weeks old) were obtained from Charles River (UK) Ltd. They were acclimatised to the animal facility for at least 5 days. Males weighed 282–408 g and females 184–239 g at study commencement. They were randomly allocated, and group-housed (4 animals/sex/treatment), under standard conditions (20–24 °C, 40–70% relative humidity, artificial light cycle 12 h light/12 h dark). Animals received SDS VRF-1 pelleted diet and drinking water ad libitum.

### Experimental design overview

All in vivo and analytical experimental segments were performed in accordance with Good Laboratory Practice standards (OECD 1998) and the adopted TK experimental design conformed to approaches set out in OECD TG 417 (2010). Comparative TK data for the various test items were obtained after single doses, and repeated dose administration for 28 days. This was based on measurement of Ag levels in whole blood at various timepoints, and also total Ag content in tissues (in the repeated dose study phase). This approach facilitated a direct quantitative comparison of parameters such as bioavailability, achieved systemic exposure and the delivered Ag dose to tissues after administration of the different Ag test items. Determination of single-dose p.o. and i.v. blood-level profiles was intended to provide information on absolute bioavailability (*F* value). A summary of the treatment groups and dosing regimens is provided in Table 2. Parallel vehicle control groups were included within each phase.

**Table 2:**
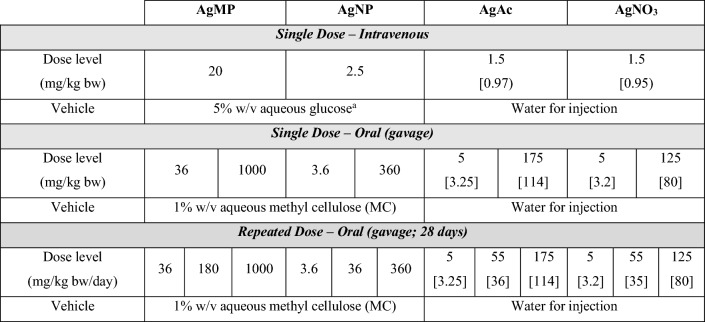
Treatment groups and dosing regimen. For each treatment, 4 male and 4 female animals were used

Parallel control groups per treatment received the respective vehicle only. Dose levels are stated as mg/kg bw with Ag equivalent dose levels for AgAc and AgNO_3_ added in square parentheses. Volume administered: oral gavage 20 mL/kg; via intravenous route either 5 mL/kg bw (AgMP and AgNP suspensions) or 2 mL/kg bw (AgAc and AgNO_3_ solutions)

^a^Isotonic glucose (5% w/v glucose in water for injection; pH adjusted to 7.0, using NaOH) has previously been demonstrated as suitable for administration of nanosilver via the i.v. route (Park and Lee 2013)

Dose levels (Table 2) were selected based on data from existing in vivo TK and toxicity studies (in the case of the soluble silver compounds and AgNP), the avoidance of overtly toxic exposures which could perturb kinetics, inclusion of intersecting matched Ag equivalent doses for the various test items, sufficient spread of treatment levels to be able to detect non-linear kinetics and anticipated analytical detection limits in biological matrices. No quantitative TK data were available for AgMP and the highest treatment level was aligned with the OECD defined Limit Dose for (sub)chronic testing.

### Dosing formulations

Polypropylene vessels and containers were used throughout preparation and storage to limit artefactual loss of Ag from adsorption onto surfaces (Sekine et al. 2015). Vessels were pre-cleaned with a dilute nitric acid solution and then rinsed thoroughly with ultrapure water to remove contaminants. Chloride- or phosphate-containing vehicles and reagents were avoided to prevent Ag precipitation. Dosing formulations were prepared once for the single-dose administrations, and weekly for the repeated dose phase (divided into daily aliquots). Further information on the pre-dose preparative procedures is given under Supplementary information. Formulations were stored protected from light, either refrigerated (2–8 °C) for AgMP and AgNP, or at ambient temperature (15–25 °C) in the case of soluble Ag test items. Preparations for i.v. administration were produced under aseptic conditions. Foundation work demonstrated that dosing formulations were homogeneous and physically stable for up to 15 days (cfr. Supplementary Information).

### Dosing procedures and standard observations

Animals (4 per sex/per treatment level) received test item via i.v. injection or oral gavage administration. Intravenous injection (bolus) was via the tail vein. Oral gavage administration was performed using a suitable graduated syringe and a rubber catheter. Dosing volumes for i.v. and oral administration were calculated based on the individual animal bodyweights. See also details provided in Table 2.

Animals were inspected visually at least twice daily for evidence of clinical signs or change in health status. As applicable, injection sites were regularly assessed post-dosing for any local reaction. Bodyweight was measured for all single-dose treatments before administration (typically the day before treatment), on the day of administration and on the day of termination. For the 28-day repeated administration phase, the bodyweight of each animal was recorded pre-treatment, on Day 1 of treatment and then at weekly intervals (including on the day of termination).

### Blood and tissue samples

Samples of whole blood (0.2 mL) were obtained from the jugular vein of animals, with lithium heparin integrated as an anticoagulant. In the single-dose study, the sample time course was 5, 15 and 30 min, and then 1, 1.5, 3, 6, 12, 24 and 48 h after dosing (i.v. treatment groups), or 1, 3, 6, 9, 12, 24, 72 and 96 h after dosing (oral treatment groups). During the 28-day repeated administration study, all animals were sampled pre-dosing and at 6 h after dosing (on Day 15), and also pre-dosing and 3, 6, 9, 12, 24, 72 and 96 h after dosing (on Day 28). Each sample was gently mixed and frozen on dry ice, pending transfer to storage at − 20 °C. After 4 week treatment, additional blood samples were obtained from animals in groups receiving AgMP and AgNO_3_ for the determination of serum copper (Cu) levels. Serum was obtained by centrifugation (10 min, 2000 RCF) and stored frozen until analysis.

Following final blood sampling, animals were euthanised using carbon dioxide asphyxiation, with subsequent exsanguination, prior to macroscopic examination for the investigation of any abnormalities. Tissues for analysis of total Ag content were obtained from animals administered the test items for 28 days. The tissue set comprised: brain (whole), bone marrow (from both femurs), small intestine (duodenum/ileum/jejenum), liver, spleen, ovaries, testes (excluding epididymides) and uterus. The luminal contents of GI tract samples were thoroughly flushed out with cold purified water. After weighing, tissues were retained frozen (− 20 °C).

### Sample processing and analysis of Ag or Cu content

Previous robust TK studies involving the analysis of Ag in biological matrices have utilised aggressive digestion techniques, typically based on digestion/mineralisation in concentrated HNO_3_ with microwave-accelerated reaction systems. To avoid subsequent precipitation of Ag into insoluble complexes and subsequent loss for analysis, sample stabilisation with an excess of chloride (e.g. HCl) is appropriate. These principles were adopted for sample processing in this investigation. In brief, each blood subsample (50 µL) was combined with 800 µL concentrated HNO_3_ (65–70%) in a glass pyrex tube. The contents were then digested in an Ultrawave (Milestone, Italy) digester using digestion programme ‘*blood*’. Afterwards, 800 μL HCl (37%) was added, and 400 μL of the HNO_3_/HCl digest was combined with 2.60 mL of rhodium internal standard followed by rotary mixing for at least 30 min. The processed sample was then analysed on an Agilent 7900 × inductively coupled plasma mass spectrometer (ICP-MS). All blood samples were analysed within 175 days (the demonstrated stability period of Ag in blood for the analytical method). For Cu analysis, a 25 µl serum subsample was added to 1.98 mL rhodium internal standard solution, mixed, and analysed by ICP-MS. Rat tissue samples were digested in HNO_3_ (65–75%) at a tissue:acid ratio of 1:3 in pyrex microwave digestion tubes. The digestion was 20 min at elevated temperature and pressure (200 °C, 100 bar, 1500 W). Then, 3 parts of concentrated HCl (35–40%) were added, and the digest was stored at − 20 °C until analysis. A 50 µL subsample was subsequently mixed with 3.95 mL of rhodium internal standard and analysed by ICP-MS. Analysis occurred within the 48-day assured stability period.

All bioanalytical methods underwent validation before deployment. ICP-MS runs included quality control samples and a multilevel calibration. Lower limits of quantitation (LLOQ) for the analytical methods were, respectively, 10 ng/mL for total Ag in blood, 5 ng/mL for total Ag in tissue and 30 ng Cu/mL for Cu in serum.

### TK parameter analysis and statistics

Descriptive statistics (means, standard deviation (SD) and coefficient of variance (CV%)) for concentration data, using appropriate grouping and sorting variables, were generated using Phoenix WinNonlin (Pharsight, Princeton, NJ). Concentration and TK parameter values were tabulated, and concentration vs. time graphs were generated. Dose effect, sex effect and time-related differences of Area Under the Curve (AUC) values were evaluated where appropriate. Concentration values below LLOQ were treated as zero for descriptive statistics. Summary statistics were not calculated if the results for at least half of the animals in a group were below LLOQ.

AUC was estimated by the linear trapezoidal rule. Dose proportionality ratios for *C*_max_ (maximum observed concentration.) or AUC_0-t_ (AUC from hour 0 to the last measurable concentration) were calculated by dividing the parameter by the corresponding value in the lower dose groups and comparing with the corresponding fold change in dose.

*T*_max_ is the time of maximum observed concentration. DN AUC is the dose-normalised AUC, calculated as $\frac{\mathrm{AUC}}{\mathrm{dose level}}$. *t*_1/2_ is the elimination half-life, determined as ${t}_{1/2}=\frac{ln2}{{\uplambda }_{z}}$ with *λ*_z_ the elimination rate constant estimated using log-linear regression during the terminal elimination phase. The number of points used in λ_z_ calculation was determined by visual inspection of the data describing the terminal phase. At least the last three time points with measurable values were used in λ_z_ calculation.

Bioavailability (*F* value) was derived from the selected AUC parameter as follows:

$$F = 100\% \times \left( {\frac{{{\text{AUC}}_{{{\text{oral}}}} }}{{{\text{AUC}}_{{{\text{iv}}}} }}} \right) \times { }\left( {\frac{{{\text{Dose}}_{{{\text{iv}}}} }}{{{\text{Dose}}_{{{\text{oral}}}} }}} \right).$$

Unless otherwise indicated, results are expressed as arithmetic group means ± SD. As applicable, one-way ANOVA was used for multiple groups followed by a Tukey–Kramer post hoc test. Statistical significance was considered at *P* < 0.05.

## Results

There were no mortalities or significant clinical signs ascribed to each of the test items. Bodyweight was also unaffected by treatment, with the exception of a slight depression in weight gain during Week 1 for animals in the repeated dose study receiving 175 mg AgAc/kg bw/d. Thereafter this group exhibited a similar trend to the control (data not shown). After repeated oral dosing, group mean organ weights were unaffected by any of the treatments (data not shown), and macropathological findings at necropsy were unremarkable aside from dark-staining of the skin and other tissues in some animals treated with the soluble Ag test items—this discoloration appeared consistent with argyria and was not associated with any adverse effect on animal health.

### TK parameters: single dose

Comparative oral route TK parameters for each of the test items—derived from time course determinations of Ag in whole blood—are presented in Table 3 (as group mean values). The Ag concentrations in all vehicle control treatments were consistently below the LLOQ (10 ng/mL for blood and 5 ng/mL for tissue).

**Table 3:** Selected TK parameters following single oral administration of the Ag test items

	TK parameters^a^	*C*_max_ (ng/mL)		*T*_max_ (h)		*t*_1/2_ (h)		AUC_(0–24)_ (ng.h/mL)		DN AUC_(0–24)_ (ng.h/mL)/(mg/kg bw/d)		AUC_(0-t)_ (ng.h/mL)		DN AUC_(0-t)_ (ng.h/mL)/(mg/kg bw/d)	
	Dose level (mg/kg bw)	M	F	M	F	M	F	M	F	M	F	M	F	M	F
AgMP	36	67.8	63.5	9	3	28.9	28.0	1150	1040	32	29	1690	1730	47	48
	1000	71.2	128	6	7.5	32.2	30.2	1130	2000	1	2	2320	4320	2	4
AgNP	3.6	25.3	70.9	4	2.5	22	24.2	424	841	118	234	406	1130	113	31
	360	254	296	9.75	9	35.2	33	4500	4800	12	13	11,200	13,500	31	37
AgAc	5 [3.25]	71.6	108	5	3.5	23.2	24.5	1050	1400	323	431	1430	2360	440	726
	175 [114]	367	632	20.3	15.3	ND^b^	25.5	6320	9970	55	87	23,300	31,100	204	273
AgNO_3_	5 [3.2]	62.5	109	2.5	3.25	18.7	30.8	994	1740	311	544	1550	4610	484	1441
	125 [80]	348	421	21	15	37.6	31.7	5970	7670	75	96	19,400	18,700	242	234

Mean values for TK parameters are reported (based on a sub-group size of 4 animals per sex; M = male, F = female). Dose levels are stated as mg/kg bw with Ag equivalent dose levels added in square parentheses for AgAc and AgNO_3_

^a^TK parameters: maximum observed Ag in whole blood concentration (*C*_max_); time of *C*_max_ (*T*_max_); calculated elimination half-time (*t*_1/2_); Area Under the Curve (AUC) and dose-normalised AUC (DN AUC) from 0–24 h [DN AUC_0-24_], and from time 0 to the last measurement [DN AUC_0-t_];

^b^*ND* Parameter not determinable

Oral route *C*_max_ values were broadly proportional to administered dose (though not monotonically), except in the case of AgMP where uptake saturation was clearly evident at 1000 mg/kg bw. Total exposure was comparable at both treatment levels, with a *C*_max_ of 64–68 ng Ag/mL at a dose level of 36 mg Ag/kg bw and 71–128 ng Ag/mL at 1000 mg Ag/kg bw. Therefore, the dose-normalised AUC_(0-t)_ values were 48 versus 2–4 (h*ng/mL)/(mg Ag/kg bw) for the low- and high-dose levels, respectively. Though not as marked in degree, less than proportional total exposure was also noted for AgNP, with dose-normalised AUC_(0-t)_ being 70–90% below predicted values (assuming linear behaviour).

Group mean *T*_max_ values for all test items were commonly resided in the range of 2.5–9 h, but *T*_max_ was significantly longer for the high-dose levels in the case of the soluble test items (15–21 h). Elimination half-life (*t*_1/2_) was comparable for AgMP and AgNP, being in the order of 22 to 35 h depending on administered dose. The *t*_1/2_ values ranged from 18 to 38 h for AgAc and AgNO_3_. In a few instances, *t*_1/2_ values were not determinable due to the lack of a distinct elimination phase (Table 3).

As absolute bioavailability (*F* values) had only been reported in the literature for a few silver compounds and nanosilver forms, a conventional single-dose bioavailability evaluation was attempted for all the test items using single administration oral and i.v. route AUC datasets. As expected, after single-dose administration, *F* values up to circa 5% were evident for AgAc and AgNO_3_ (data not shown). However, highly anomalous results were obtained in the case of AgMP in relation to blood concentration profiles following i.v. bolus administration. Resultant *F* values were clearly contrasting the AUC data via the oral route after a single dose. Separate mass-balance calculations indicated that very rapid clearance of i.v. administered AgMP from the blood compartment had occurred, with high inter-individual variability. Dose-normalised AUC group mean values provided an alternative comparator of achieved systemic exposure after single oral exposure to each of the test items (Table 3). Based on these data, when comparing AgMP with soluble Ag compounds, the achieved systemic exposure was 10- to 30-fold lower in the case of the lower dose comparators, and it ranged from approximately 40- to 100-fold less for high-dose region comparators. Appreciably greater systemic availability was demonstrated for AgNP than AgMP. However, it was still less than AgAc and AgNO_3_; i.e. circa two-to-fivefold or five-to-eightfold lower for low- and high-dose comparators, respectively. In terms of gender-specific TK differences, in the case of the soluble Ag compounds and AgNP, DN AUC were relatively higher for female animals at the lowest treatment levels (i.e. where appreciable saturation of kinetics is not expected). No such difference was apparent for animals which received AgMP.

### TK parameters: repeated dosing

For all test items, the group mean pre-dose and 6 h post-dose blood Ag concentrations evident on Day 15 were generally similar to those on Day 28 (Day 15 data not shown) which indicated that steady-state kinetics had been achieved by 2 weeks of repeated oral dosing. This finding is comparable with estimates of time to steady-state conditions from previous reports relating to both soluble silver compounds and various types of AgNP (e.g. van der Zande et al. 2012).

Derived TK parameters following 28 days repeated oral dosing are summarised in Supplemental Table S1. These were broadly consistent with those obtained from the single-dose administration experiment. Noted exceptions were: an anticipated higher *C*_max_ evident by Day 28 for all test items, a shorter *T*_max_ in the case of the AgAc high-dose group and generally higher *t*_1/2_ values when comparing results for low- and high-dose groups between Day 28 versus Day 1. Most calculated t_1/2_ values after 28 days of dosing resided in the range of 30–40 h, with no clear differences evident between the various test items. In the repeated administration experiment, sex differences in *C*_max_ and AUC_(0-t)_ were typically less than twofold with values for females being uniformly greater than for males (i.e. a pattern consistent with that observed following administration of a single dose).

**Fig. 1:**
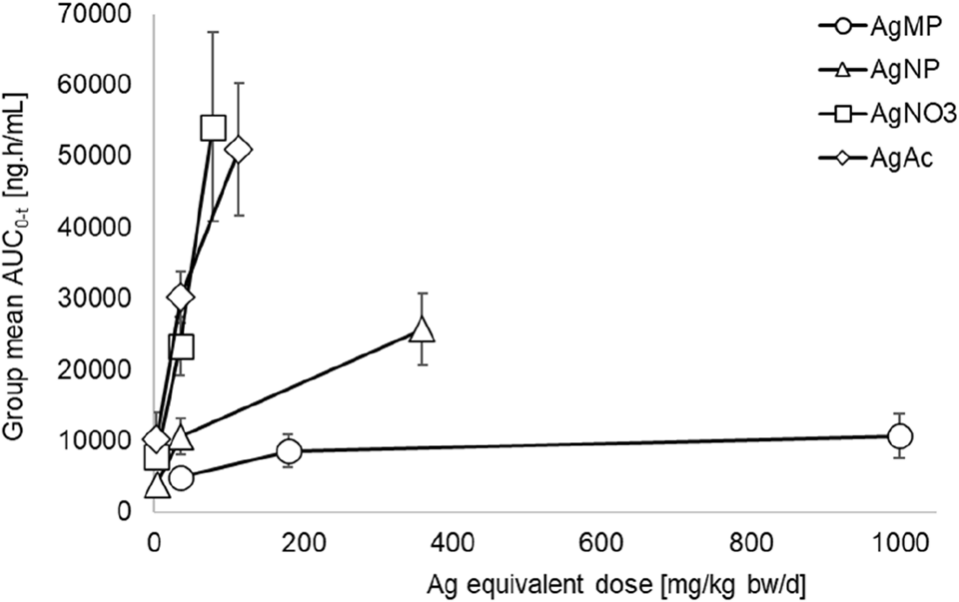
Achieved systemic exposure for soluble Ag compounds (AgAc and AgNO_3_), AgNP or AgMP following 28 days repeated dosing via oral gavage. Depicted values are group mean AUC_0-96 h_ values (± Standard Deviation) for female animals plotted against Ag equivalent dose (mg Ag/kg bw/d). Data for males were closely comparable in terms of differential patterns (not shown)

From AUC and *C*_max_ values, it was again evident that the extent of absorption attained for AgAc and AgNO_3_ was equivalent (Fig. 1; Table 4; Table S1). In notable contrast, based on AUC determinations, marked differences existed in the extent of absorption observed for these soluble Ag compounds compared to that for AgMP with the degree of systemic exposure achieved being considerably lower for AgMP (Fig. 1 and Table 4). For instance, when AUC values obtained for directly matched Ag equivalent treatment levels were examined (e.g. AgAc intermediate-dose group versus AgMP low-dose group), systemic exposure was six-to-eightfold lower in the case of AgMP (Table 4). Due to the non-linear kinetics evident for AgMP, this differential became more marked when dose-normalised AUC values at the highest tested treatment levels were considered (Table 4). Under these circumstances, the fold-difference was 30- to 60-fold less for AgMP versus the soluble Ag compounds. Unlike the linear kinetics evident for AgAc and AgNO_3_, uptake of AgMP was non-linear as the amount administered was increased up to a limit dose, with plateauing of absorption being evident for this test item (Fig. 1).

**Table 4:** Group mean area under curve from time 0 to the last measurement 96 h post-dose (AUC_0-t_) and corresponding dose-normalised AUC (DN AUC_0-t_) values following 28 days repeated dosing via oral gavage

AgAc	AUC_0-t_ (ng.h/mL)		DN AUC_0-t_ (ng.h/mL)/(mg/kg bw/d)		AgNO_3_	AUC_0-t_ (ng.h/mL)		DN AUC_0-t_ (ng.h/mL)/(mg/kg bw/d)	
Dose level	M	F	M	F	Dose level	M	F	M	F
5 [3.25]	6220	10,200	1770	2960	5 [3.2]	5470	7690	1600	2240
55 [36]	23,800	30,200	664	846	55 [35]	22,600	23,300	581	601
175 [114]	43,000	51,000	342	400	125 [80]	42,800	54,100	476	601

AgNP	AUC_0-t_ (ng.h/mL)		DN AUC_0-t_ (ng.h/mL)/(mg/kg bw/d)		AgMP	AUC_0-t_ (ng.h/mL)		DN AUC_0-t_ (ng.h/mL)/(mg/kg bw/d)	
Dose level	M	F	M	F	Dose level	M	F	M	F
3.6	2470	3900	686	1080	36	2740	4850	82	145
36	7070	10,600	186	276	180	5980	8550	36	51
360	13,400	25,700	35	67	1000	7130	10,700	9	13

Dose levels are stated as mg/kg bw/d with Ag equivalent dose levels added in square parentheses for AgAc and AgNO_3_. Sub-group size = 4 animals per sex (M = male, F = female)

The degree of systemic exposure apparent for AgNP was greater than that for AgMP. For all treatment levels, it was intermediate between AgMP and AgAc / AgNO_3_ (Fig. 1; Table 4).

### Tissue Ag levels and distribution patterns

After administration of the test items for 28 days, Ag was detectable in most tissues (Table S2). In terms of tissue distribution patterns, the spleen and GI tract were identified as major sites of distribution for both sexes, with lower but still notable absolute Ag concentrations being evident in the ovary, liver, and uterus, particularly after treatment with AgAc and AgNO_3_ (Table S2). The brain and testis were minor sites of distribution, with the maximal Ag concentrations in these tissues showing evidence of plateauing as treatment levels increased (Table S2). For bone marrow, there were technical issues with the consistency of obtained sample size which complicated overall interpretation. For this tissue, Ag concentrations from animals treated with AgMP and AgNP were typically below LLOQ, but absolute Ag concentrations were moderately high for animals treated with AgAc and AgNO_3_.

**Fig. 2:**
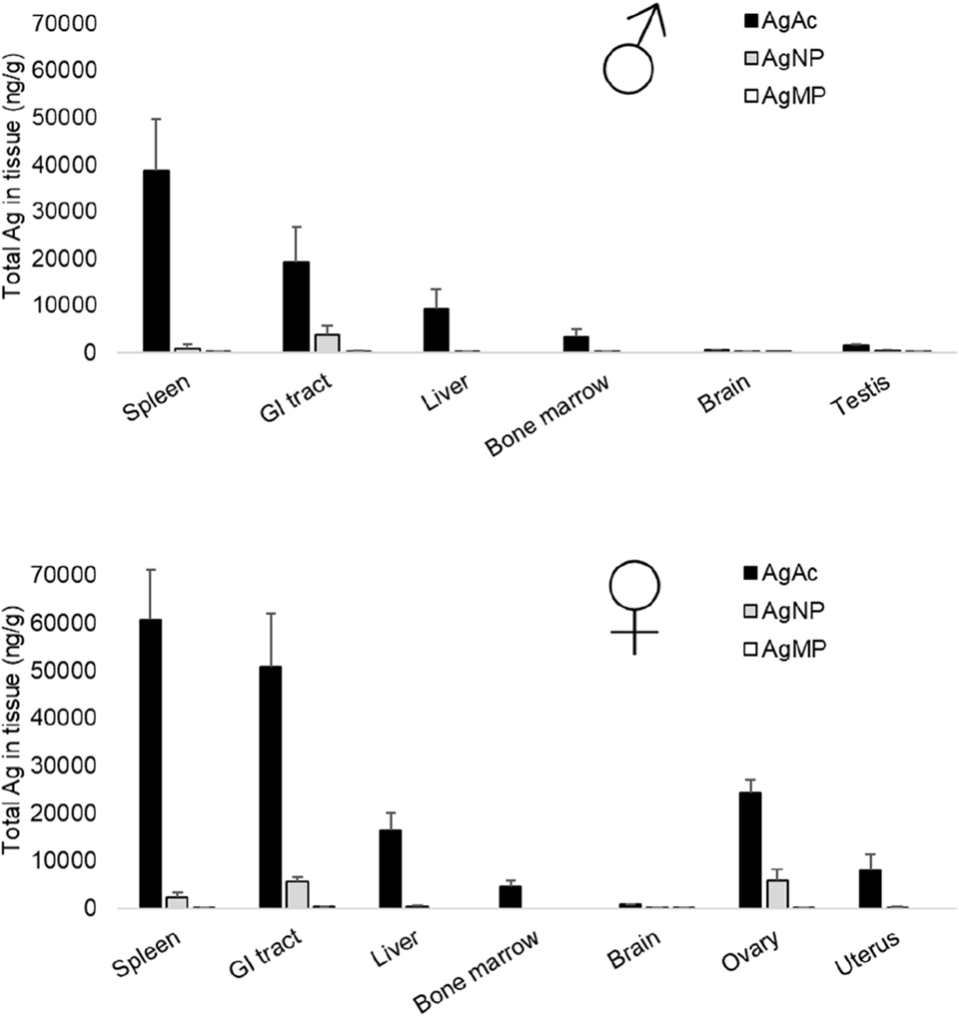
Ag in tissue concentrations for silver acetate, nanosilver and silver powder following 28 days repeated dosing via oral gavage. Data series shown are at a matched Ag equivalent treatment level for the respective test items (35–36 mg Ag/kg bw/d). Mean values (*n* = 4/sex) and standard deviation for male (top figure) and female (bottom figure) animals are reported, including the sex-specific tissues ovary, uterus and testis. Ag concentrations were significantly higher in all tissues for silver acetate-treated animals compared to those receiving nanosilver or silver powder (*p* < 0.05). Except for spleen of male animals, Ag concentrations were significantly higher in all tissues for nanosilver-treated animals compared to those receiving silver powder (*p* < 0.05)

Silver concentrations increased in a dose-dependent manner, although there were clear differences between the various tissues, and in the differential profiles of the different test items (Fig. 2; Table S2). In general, absolute tissue concentrations were highest for AgAc and AgNO_3_, with levels after AgMP treatment being substantially lower, whilst the profile for AgNP was intermediate. To exemplify the degree of difference evident between the AgMP and AgNP: at a matched Ag equivalent dose level (36 mg Ag/kg bw/d), the achieved tissue exposures were typically an order of magnitude higher in the case of the AgNP than for AgMP. In male and female reproductive tract tissues, the detected Ag levels were 6 to 73-fold higher for AgNP than for AgMP (at this same dose-normalised comparator exposure). For AgMP administered at limit dose (1000 mg/kg bw/d), group mean absolute Ag concentrations in the principal organs of distribution (spleen and GI tract) were in the low ppm level, and typically an order of magnitude lower (ppb range) in other tissues examined.

### Serum Cu pools

**Fig. 3:**
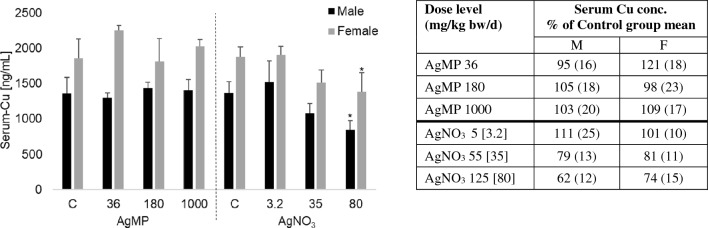
Serum Cu status (in ng Cu/mL) of rats orally exposed to silver powder (AgMP) or silver nitrate (AgNO_3_) following 28 days repeated dosing via oral gavage. Mean values (*n* = 4/sex) and standard deviation for male (black) and female (grey) animals are reported. Significantly different serum Cu level from control is depicted with *(*p* < 0.05). Inset table: relative serum Cu concentrations compared to the control for male (M) and female (F) animals. Values are mean values (*n* = 4/sex) with corresponding standard deviation between brackets. Dose levels are stated as mg/kg bw/d with Ag equivalent dose levels added in square parentheses for AgNO_3_

Serum Cu concentrations in control animals corresponded with the expected levels for the SD rat tester strain at about 1400 or 1900 ng Cu/mL for males and females, respectively. A reduction in circulating Cu levels was observed in animals treated with AgNO_3_, with a detectable depression at 55 mg/kg bw/d (35 mg Ag/kg bw/d) and being statistically significant at 125 mg/kg bw/d (80 mg Ag/kg bw/d). This corresponded to a depression of serum Cu to 62% or 74% of control mean values for male and female subgroups, respectively (Fig. 3). In the case of animals treated with AgMP for 28 days, there was no indication of any depletion in circulating Cu levels (Fig. 3), even at limit dose (1000 mg/kg bw/d, i.e. a Ag equivalent exposure 12 times greater than that of the AgNO_3_ high-dose group).

## Discussion

### Ag levels in blood

To our knowledge, this study is the first systematic investigation comparing the TK profiles of soluble Ag compounds (AgAc and AgNO_3_), Ag nanoparticles and a ‘bulk’ Ag form (fine Ag powder). The main findings for the oral bioavailability of soluble Ag compounds and AgNP, elucidated from AUC measurements of Ag in blood, were congruent with the previous reports using rodent models (Bachler et al. 2013; Boudreau et al. 2016; Loeschner et al. 2011; Park 2013; van der Zande et al. 2012). Hence, the reference soluble compounds selected for this study (AgAc and AgNO_3_) were shown to be the most bioavailable forms following either single dose or repeated administration to adult rats, with a small-sized AgNP (15 nm) exhibiting rather lower bioavailability (Tables 3 and 4; Fig. 1). In contrast, AgMP—a micron-size Ag powder—was much less absorbed via the oral route, with achieved systemic exposure being 10- to 100-fold lower than soluble Ag compounds after a single dose and a similar differential pattern being evident following repeated administration for 28 days. Lower absolute Ag in tissue concentrations observed for AgMP when compared to those evident for soluble Ag compounds (Table S2; Fig. 2) further support that conclusion. Whilst silver in blood AUC values (repeated dosing) for AgAc and AgNO_3_ remained linear with increasing dose for all treatment levels, an early and obvious downward inflection of AUC values towards non-linear kinetics occurred with AgMP. This likely represents the saturation of one or more rate-constrained Ag^+^ release processes from AgMP particles within the GI tract. The very limited dissolution characteristic of the AgMP test item and the minimal Ag^+^ fraction typically associated with such particulate Ag forms (Supplemental Information; Table 1) are considered to be key determinants of its TK profile.

The attempt to derive an absolute bioavailability value (F value) for AgMP was impeded by a very rapid decrease in Ag in blood concentrations following a single i.v. bolus dose of the AgMP suspension. It is known that certain micron-size particulates, including metals, can be rapidly sequestered by the reticuloendothelial system when intravenously administered, leading to circulatory half-times in the order of a few minutes (Neuberger et al. 2005; Liu et al. 2013). Also, reaction of i.v. administered particles with blood proteins or other biomolecules might form a corona around the particles and affect particle dissolution, transport, and retention in the blood and blood vessels (Recordati et al. 2016). These considerations call into question the validity of applying the conventional *F* value derivations in the case of elemental Ag particulate forms.

### Ag levels in tissue

Dependent on the Ag test item administered in the 28-day TK segment, there were marked differences in absolute Ag concentrations in the tissue set investigated. However, the pattern of tissue distribution was qualitatively similar for all the test items, the rank order being spleen > GI tract > ovary > liver > uterus > bone marrow > testis≈brain (in the case of AgMP, the order for GI tract and spleen was reversed). As the site of first contact in an oral route study, significant uptake into GI tract is expected, as shown in prior TK studies with soluble silver compounds and Ag nanoparticles. Our results also align with reports in rodent models that reticuloendothelial organs, viz., spleen and liver, are important sites of distribution (e.g. van der Zande et al. 2012). From absolute tissue Ag levels, it is clear that the testis and brain are minor sites of distribution (particularly in the case of soluble Ag compounds). Plateauing of Ag concentrations was evident in both tissues as the dose level increased. This is expected if blood–testis and blood–brain barriers operate to limit uptake of Ag^+^, as demonstrated for brain where identifiable Ag depots are associated with the CNS endothelial sub-compartment or are deposited in basal lamina of the choroid plexus (Rungby and Danscher 1983a,b). Similar saturation kinetics, as well as identification of these tissues as minor sites of distribution, have been shown in several previous TK studies in rodent models (by oral and parenteral routes of administration); e.g. Loeschner et al. 2011; Lankveld et al. 2010; Kim et al. 2008; Pang et al. 2016; Juling et al. 2016; Gan et al. 2020. Other reports concluding that the brain and testis represent significant sites of Ag deposition were based on administration of Ag^+^ or AgNP solely at low doses below the uptake saturation thresholds. It remains to be determined whether the primary mode of uptake of Ag into the testis and brain is by Ag^+^ ion active transport, though rate-limited metal transporter systems are known to exist within the blood-organ barriers. An example is copper transporter 1 (CTR1) which modulates copper transport to testicular germinal cells and Sertoli cells (Herman et al. 2020), bearing in mind Cu(I) and Ag(I) are isoelectronic with the same d-shell electronic configuration, have similar ionic radii and soft acid behaviour, and—as later discussed—that Ag^+^ is an interchangeable substrate for this transporter (Bertinato et al. 2010).

This study provides gap-filling data for the female reproductive system, given that no reliable data previously existed for ovarian distribution, and information on uterine levels was fragmentary. As detailed, it has been demonstrated here that the ovary is an important site of distribution. Due to this finding, ancillary studies were performed to speciate the nature of the intra-organ Ag deposits (cfr. Supplementary Information). The ovary has a particular intra-organ environment which favours the sequestration of systemically available Ag^+^, via its conversion into Ag-sulphur/selenide complexes. Such stable Ag–S–Se complexes possess ultralow solubility (K_sp_ values of ~ 10^–50^ to 10^–65^), thus exhibiting very low local bioavailability. They are the dominant chemical species in tissue argyria, which has been recognised as the key mammalian detoxification mechanism for the Ag ion (Lansdown 2010; Aaseth et al. 1981; Berry and Galle 1982).

### Ag toxicokinetics

Based on the data from the single and repeat dose TK experiments, including results for Ag deposition in tissues, AgNP exhibited a TK profile which was intermediate between the soluble Ag compounds and AgMP (Fig. 1). Most published studies have indeed shown that AgNP is less bioavailable via the oral route than soluble silver compounds such as AgNO_3_ or AgAc. Though, dependent on their specific characteristics, the degree of oral absorption may still be significant (van der Zande et al. 2012; Boudreau 2012; Bachler et al. 2013; Park and Lee 2013; Park 2013; Loeschner et al. 2011). Smaller size AgNP are typically more bioavailable (Boudreau et al. 2016), an observation compatible with the influence of the surface area-to-size relationship and the propensity for oxidative dissolution of Ag^+^ as it relates to diminishing particle size (Liu et al. 2012; Batchelor-McAuley et al. 2014). Information is partial and conflicting as to whether capping systems, such as citrate or polyvinylpyrrolidone (PVP) ligands, have a substantial effect on the oral or parenteral bioavailability of AgNP (Pang et al. 2016; Bachler et al. 2013). However, a potentially much more impactful variable is that nearly all AgNP have an associated free Ag^+^ fraction, which in some cases can be quantitatively substantial and increase on ageing to comprise 40% or more of the total Ag content (Kittler et al. 2010; van der Zande et al. 2012). This may provide a basis for why some TK studies in the literature have described bioavailabilities for AgNP close to those of soluble Ag compounds, and why inconsistent observations for ostensibly similar AgNP have been described. Outcomes from a well-conducted oral TK study by van der Zande and co-workers (2012) support this postulate. For two types of AgNP with differing stabilisation system (but equivalent size), Ag concentrations in organs were reported as being highly correlated to the amount of Ag^+^ in the nanoparticulate formulation but not to the type of capping system. With all the aforementioned confounders in mind, we selected a AgNP test item corresponding to a well-characterised certified reference nanomaterial (CRM), with a low and stable Ag^+^ fraction (~ 5% of total Ag content). Use of a AgNP with a capping system prone to Ag^+^ liberation was also avoided, and a stable dissolution profile was confirmed in the dosing vehicles (cfr. Supplementary Information).

Other aspects of the TK profiles of the various Ag test items corresponded to previously established characteristics. For instance, calculated *t*_1/2_ for the soluble Ag compounds and AgNP were consistent with results from other reliable studies performed in rat TK models (Boudreau et al. 2016; Park et al. 2011; van der Zande et al. 2012). Comparison of day 15 and terminal blood concentrations of Ag indicated that steady-state kinetics were achieved by about 2 weeks, which corresponds with a previous investigation (van der Zande et al. 2012). Irrespective of the Ag test item administered, slightly higher systemic exposure was attained in female animals (i.e. circa 1.5-fold; Table 4), which is again qualitatively consistent with previous rodent studies (Boudreau et al. 2016; Kim et al. 2008). This difference was also mirrored in terms of gender-specific achieved tissue dose (Table S2). The basis for this gender-related variation in biokinetics remains to be established.

### Ag interference with serum Cu

A principal mechanism involved in the toxicity of Ag^+^ is the induction of secondary Cu deficiency, which may lead to a variety of adverse haematological, biochemical and developmental effects (Shavlovski et al. 1995; Sprando et al. 2017; Renaut 2022). Ag^+^ causes structural deformation of the carrier protein ceruloplasmin, interfering with its Cu-binding capacity (Hirasawa et al. 1997; Ilyechova et al. 2014), although Ag^+^ may also interfere with key cellular Cu transporter systems, including CTR1, since Ag^+^ is interchangeable with Cu^+^ (Lee et al. 2001; Zimnicka et al. 2007). To date, the mammalian toxicity information available for ‘bulk’ Ag forms (like Ag powder) is extremely limited, and no data exist on its potential to induce Cu deficiency. Serum Cu measurements following repeated exposure of rats to AgMP for 28 days demonstrated no reduction in circulating total Cu levels up to limit dose of 1000 mg Ag/kg bw/d (Fig. 3). This contrasted with findings for AgNO_3_ (a prototypic soluble Ag compound), whereby a statistically significant Cu depletion was evident (a comparable interference was identified for AgAc by Renaut 2022). The absence of effects for AgMP is very likely a reflection of its low oral bioavailability, including the correspondingly minor concentration of Ag delivered into the blood compartment. At toxicologically relevant dose levels, ‘bulk’ Ag forms like AgMP are predicted to have negligible potential to produce indirect toxicity via perturbation of Cu homeostasis.

## Conclusion

From systemic exposure indicators (Ag in blood results), together with Ag in tissue data, it is concluded that the relative oral bioavailability of the various forms of silver evaluated was AgAc = AgNO_3_ > AgNP >  > AgMP. The AgMP test item in this study represents one of the smallest powders in widescale industrial use and is predicted to be a conservative biokinetics representative for other ‘bulk’ forms available in commerce (i.e. for powders of comparable or larger dimensions as well as massive Ag forms). Part of the applicability of our work relates to substance grouping and read-across as they pertain to mammalian toxicity data (systemic endpoints) for various silver test items. Based on the TK profile divergence described here (including blood Ag and tissue Ag profiles), the direct read-across of toxicity data obtained for either soluble Ag compounds or nanosilver to ‘bulk’ silver forms (like powders or massives) is not supported. Definitive judgements for nanosilver grouping and read-across remain a challenge, as significant variability in absorption characteristics has been described between various types of AgNP. This may be attributable to large differences in co-existent Ag^+^ fraction associated with a particular AgNP, as well as size and surface chemistry variables (all of which are insufficiently studied in comparative in vivo TK investigations). From a TK perspective, it may be justified to group certain AgNP together with soluble Ag compounds, for instance where the former contains substantial-free Ag^+^ fractions or have dissolution characteristics making them similar to soluble Ag compounds.

## Supplementary Information

Below is the link to the electronic supplementary material.

Supplementary file1 (DOCX 1695 KB)

## Data Availability

Data and materials are available from the authors upon reasonable request.
