# Supplementary material for: Comparative in vivo toxicokinetics of silver powder, nanosilver and soluble silver compounds after oral administration to rats
Source: Arch Toxicol. 2023 May 17;97(7):1859–72. doi: 10.1007/s00204-023-03511-6 (PMC10256634; doi:10.1007/s00204-023-03511-6)
Supplement: Supplementary file 1 — Supplementary file1 (DOCX 1695 KB) [file 204_2023_3511_MOESM1_ESM.docx]

**SUPPLEMENTARY INFORMATION**

**Characterisation of test items within dosing formulations.**

An extensive analytical pre-testing program was performed to fully characterize the test items and develop protocols to optimise the homogeneity and stability of dosing formulations. Details of this program are separately available (Tirez et al. 2020). The enabling work addressed the characterisation in dosing vehicles of the suspension test items (i.e., AgNP and AgMP), which are more challenging to formulate in terms of dispersion, agglomeration and sedimentation aspects. The principal techniques employed were X-ray diffraction spectroscopy, Dynamic Light Scattering (DLS), Scanning Electron Microscopy (SEM), Scanning Transmission Electron Microscopy (STEM), and filtration techniques coupled to either Inductively Coupled Plasma-Mass Spectrometry (ICP-MS) or Inductively Coupled Plasma-Atomic Emission Spectrometry (ICP-AES). The ionic silver (Ag^+^) fraction in the nanosilver test item (AgNP), as supplied, was 5% (of total Ag), and was maintained at or below this level when prepared in the dosing formulation. The Ag^+^ fraction in the silver powder (AgMP) dosing formulations was determined to be a maximum of 0.004%. Ag^+^ fractions in the selected dosing formulations remained stable for an acceptable storage period (up to 11 days). This enabling work confirmed that no major precipitation, dissolution, agglomeration/aggregation or other unexpected physico-chemical events occurred during dosing formulation and subsequent storage which might impact the TK study results.

1% w/v aqueous methyl cellulose (MC) as well as aqueous glucose were unsuitable as dosing vehicle selections for the soluble silver test items (AgAc and AgNO_3_). In the case of both vehicles, total dissolved Ag concentrations in AgAc and AgNO_3_ test formulations altered substantially over time, in some cases with the appearance of obvious precipitation, colour change or solution opacity. This was likely caused by reduction reactions leading to respeciation of dissolved Ag^+^ to insoluble Ag^0^. Therefore, purified water was selected as alternative vehicle for these test items. It was demonstrated to provide satisfactory physical stability and homogeneity of formulations for the required periods of use and storage.

**Dosing formulations - suspension preparative (pre-dosing) stages.**

Suspension formulations of silver powder (AgMP) or nanosilver (AgNP) in vehicle for i.v. administration (5% w/v glucose in water at pH 7.0) were prepared by taking the required amount of test item and adding about 50% of the final volume of vehicle to the container. The mixture was magnetically stirred followed by 1-2 minutes sonication. The mixture was then moved to a final container, the weighing vessel was rinsed with the vehicle and the rinsings added. The total volume was corrected, and the mixture was magnetically stirred for minimum 20 minutes.

Suspension formulations of AgMP and AgNP in vehicle for oral administration (1% w/v aqueous methylcellulose [400 cps]) were prepared by adding the required amount of test item to 50% of the final volume of vehicle, whilst magnetically stirring at an appropriate speed to obtain a vortex which was half the depth of the suspension. The weighing container was rinsed thoroughly and the rinsings added. Then the suspension was stirred for 1 minute, and subsequently sonicated for approximately 30 minutes (this stirring and sonication procedure being repeated once more). Afterwards, the stirring continued at an appropriate speed to obtain a vortex (half the depth of the final suspension) for at least 60 minutes prior to administration.

**Dosing formulations - achieved concentration, stability and homogeneity.**

To assure their accurate concentration, uniform homogeneity and physical stability, dosing formulation concentrations were analytically verified after preparation but before first administration using inductively coupled plasma mass spectrometry (ICP-MS) for determination of total Ag content. The analytical methodology having first been validated under GLP conditions to analytical procedure integrity norms. Samples of each formulation prepared for i.v. and single oral administration, as well as for Weeks 1 and 4 of the repeated dosing segment were analysed. In brief, 1 mL subsamples were taken in triplicate and 4 mL of concentrated HNO_3_ (analytical grade) was added. Samples were stored at ambient temperature protected from light prior to analysis. Dosing formulation subsamples (25 µL) were added to 9.98 mL of rhodium internal standard solution, capped and rotary mixed for at least 30 minutes. An aliquot of each processed sample was then analysed by ICP-MS, together with quality control samples and a multilevel calibration.

Acceptance criteria for formulations containing dissolved test items were set at ±10% of nominal concentrations, or else ±15% of nominal concentrations in the case of suspension forms. In general, dosing formulation analyses were within acceptance criteria, and no Ag was detected in the vehicle controls. Isolated instances occurred where measured Ag concentration was slightly outside the defined acceptance limits, although replicate variability was tolerable. These few exceptions were judged not to have a material impact on the overall integrity of the study.

**Localisation and characterisation of Ag in ovary.**

Results from the repeat dosing TK study demonstrated that the ovary was a significant site of Ag distribution, in terms of relative rank order of tissue distribution for all the test items, and in terms of the notable absolute Ag in tissue concentrations detected for the soluble Ag compounds (AgAc and AgNO_3_) and for AgNP. The ovary is known to contain relatively high levels of sulphur and selenium, undergoing more rapid turnover than in some other tissues (Boja et al. 2005; Ceko et al. 2016; Thomson et al. 1975). We hypothesised that such an intra-organ environment could favour sequestration of systemically available Ag^+^, via its conversion into stable Ag sulphur/selenide complexes. Therefore, ancillary investigations were performed on ovarian tissue obtained from rats treated with AgAc in order to obtain information on the speciation of the Ag depots. This work, based on Scanning Electron Microscopy-Energy Dispersive X-Ray Spectroscopy (SEM-EDX) characterisation directed by autometallography (AMG) localisation, confirmed that the ovarian Ag depots were associated with sulphur- and selenium-containing moieties as 250-500 nm size structures present in the tissue (refer to representative micrographs in Fig. S1; Lison and Ryelandt 2021). This type of co-location pattern is strongly indicative of the existence of Ag sulphide/selenide complexes. Many previous studies have identified similar tissue-fixed complexes as the key chemical species which form the basis of argyria – a phenomenon commonly recognised as a key mammalian detoxification mechanism for Ag^+^ (Mota and Dinis-Oliveira 2021). It is known that such Ag sulphur/selenide complexes possess ultralow solubility with very low mobility and bioavailability locally in tissues. Support for the postulate that selenium-containing complexes may be involved (as well as Ag-sulphide-based species) arises from tissue selenium determinations. Treatment with a soluble silver compound (AgAc) has been shown to elevate total ovarian Se levels markedly above control values (Renaut et al. 2022 [unpublished results]) – a finding indicative of trapping of Se within the immobile complexes.

**References**

Boja ES, Hoodbhoy T, Garfield M, Fales HM (2005) Structural conservation of mouse and rat zona pellucida glycoproteins. Probing the native rat zona pellucida proteome by mass spectrometry. Biochemistry 44: 16445-16460. https://doi.org/10.1021/bi051883f

Ceko MJ, O'Leary S, Harris HH, Hummitzsch K, Rodgers RJ (2016) Trace Elements in Ovaries: Measurement and Physiology. Biol Reprod. 94:1-14. https://doi.org/10.1095/biolreprod.115.137240

Lison D and Ryelandt L (2021) Scanning electron microscopy (SEM)-energy dispersive X-ray (EDX) studies of ovarian tissue obtained from rats treated with a form of ionic silver (silver acetate). LTAP, University of Louvain. <https://www.epmf.be/scipub/> (accessed 2 March 2023)

Mota L and Dinis-Oliveira RJ (2021) Clinical and forensic aspects of the different subtypes of argyria. J Clin Med. 10:2086. https://doi.org/10.3390/jcm10102086

Thomson CD, Robinson BA, Stewart RD, Robinson MF (1975) Metabolic studies of [^75^Se]selenocystine and [^75^Se]selenomethionine in the rat. Br J Nutr. 34:501-509. <https://doi.org/10.1017/S0007114575000542>.

Tirez K, Smets L, Beutels F, Mertens M, Kemps R, Verheyde B, Velimiromvic M (2020) Physico-chemical characterisation of micron-sized silver powder and nano silver suspension. Final report 2020/Unit SCT/R/2168 to European Precious Metals Federation, VITO NV, Mol, Belgium. <https://www.epmf.be/scipub/> (accessed 2 March 2023)


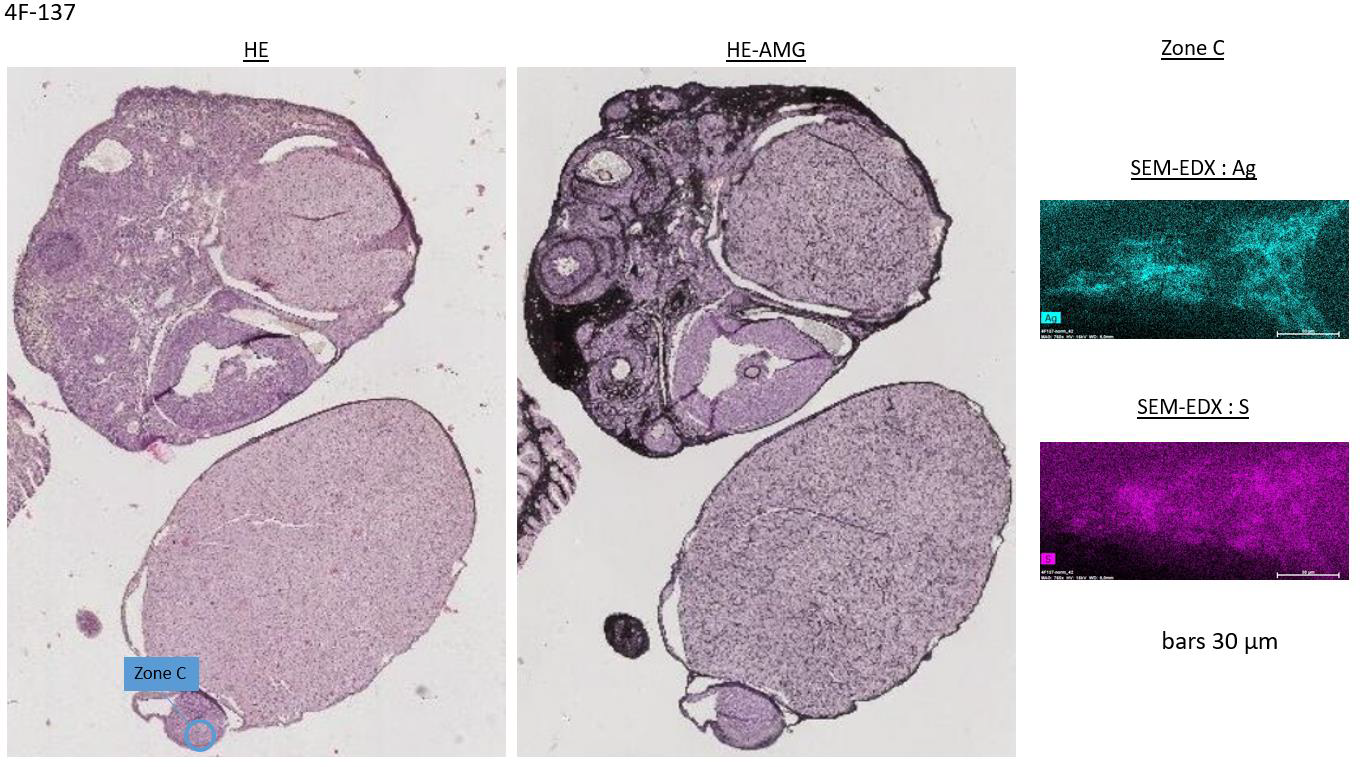


**Fig. S1** Micrographs of an ovarian tissue section from an animal exposed to repeated oral doses of AgAc. Ovarian tissue was obtained from a young adult Sprague Dawley rat which received 160 mg/kg bw/d AgAc (p.o.) for 8 weeks during a separate preliminary reproductive toxicity study. Representative of images obtained from a total of 3 animals. From left to right: Optical microscopy (H&E staining); Ag-autometallography (AMG) image; SEM-EDX images with element cartography.

**Table S1** Selected TK parameters^a^ determined after 28 days repeated oral dose administration. Mean values for TK parameters are reported (based on a sub-group size of 4 animals per sex). Dose levels are stated as mg/kg bw with Ag equivalent dose levels added in square parentheses for AgAc and AgNO_3_.

|  | **Dose level (mg/kg bw/d) [Ag equivalent dose]** | **C_max_**  **(ng/mL)** | | **T_max_**  **(h)** | | **t_1/2_**  **(h)** | | **DN AUC_(0-24)_**  **(ng.h/mL)/**  **(mg/kg/d)** | | **DN AUC_(0-t)_**  **(ng.h/mL)/**  **(mg/kg/d)** | |
| --- | --- | --- | --- | --- | --- | --- | --- | --- | --- | --- | --- |
|  |  | **M** | **F** | **M** | **F** | **M** | **F** | **M** | **F** | **M** | **F** |
| **AgMP** | 36 | 73.3 | 125 | 6 | 4.5 | 29.3 | 36.6 | 38.2 | 63.3 | 82.4 | 145 |
|  | 180 | 123 | 192 | 4.5 | 3 | 33.4 | 41.4 | 14.2 | 21.6 | 36.1 | 50.9 |
|  | 1000 | 139 | 229 | 4.5 | 6 | 31.5 | 43.4 | 3.39 | 5.39 | 8.85 | 13.1 |
| **AgNP** | 3.6 | 62 | 90.8 | 6 | 3 | 31.7 | 36.4 | 337 | 431 | 686 | 1080 |
|  | 36 | 150 | 278 | 6 | 6 | 36.9 | 38.9 | 76.6 | 118 | 186 | 276 |
|  | 360 | 299 | 485 | 9 | 10.5 | 42.4 | 40.7 | 14.3 | 26.0 | 35.3 | 67.3 |
| **AgNO_3_** | 5 [3.2] | 132 | 177 | 4.5 | 6 | 42.1 | 37.5 | 670 | 940 | 1600 | 2240 |
|  | 55 [35] | 413 | 483 | 12 | 10.5 | ND^b^ | 45.0 | 212 | 244 | 581 | 601 |
|  | 125 [80] | 738 | 997 | 12 | 18 | 42.0 | 39.8 | 166 | 227 | 476 | 601 |
| **AgAc** | 5 [3.25] | 153 | 216 | 7.5 | 3 | 32.1 | 41.3 | 741 | 1130 | 1770 | 2960 |
|  | 55 [36] | 404 | 477 | 6 | 6 | ND | 46.8 | 228 | 280 | 664 | 846 |
|  | 175 [114] | 766 | 930 | 3 | 3 | 36.3 | 40.4 | 121 | 152 | 342 | 400 |

^a^TK parameters: maximum observed Ag concentration in whole blood (C_max_); time of C_max_ (T_max_); calculated elimination half-time (t_1/2_); Dose Normalised Area Under the Curve from time 0 to 24 h post-dose (DN AUC_0-24_) and time 0 to the last measurement at 96 h (DN AUC_0-t_).

^b^ND = Parameter not determinable.

**Table S2** Silver levels in selected tissues (ng Ag/g tissue) after 28 days repeated oral dose administration. Mean tissue levels are reported with standard deviation (SD) (based on a sub-group size of 4 animals per sex). Dose levels are stated as mg/kg bw with Ag equivalent dose levels added in square parentheses for AgAc and AgNO_3._

|  | **Dose level (mg /kg bw/d)** | **Spleen** | | | | **Bone marrow** | | | | **Brain** | | | | **Liver** | | | |
| --- | --- | --- | --- | --- | --- | --- | --- | --- | --- | --- | --- | --- | --- | --- | --- | --- | --- |
|  |  | **M** | | **F** | | **M** | | **F** | | **M** | | **F** | | **M** | | **F** | |
|  |  | Mean | SD | Mean | SD | Mean | SD | Mean | SD | Mean | SD | Mean | SD | Mean | SD | Mean | SD |
| **AgMP** | 36 | 78 | 31 | 90 | 59 | <LLOQ |  | <LLOQ |  | 76 | 16 | 76 | 18 | <LLOQ |  | 77.9 | 34 |
|  | 180 | 164 | 141 | 1006 | 623 | NQ |  | <LLOQ |  | 117 | 47 | 166 | 35 | 64.3 | 30 | 405 | 619 |
|  | 1000 | 516 | 195 | 1448 | 830 | NQ |  | 243 | 89 | 159 | 26 | 187 | 28 | 48.7 | 9 | 274 | 186 |
| **AgNP** | 3.6 | 77 | 39 | 53 | 22 | <LLOQ |  | <LLOQ |  | <LLOQ |  | 65 | 13 | <LLOQ |  | 77.2 | 50 |
|  | 36 | 938 | 920 | 2233 | 1069 | 181 | 107 | NQ |  | 226 | 47 | 221 | 13 | 154 | 114 | 382 | 233 |
|  | 360 | 14928 | 5257 | 28081 | 7076 | NQ |  | NQ |  | 575 | 137 | 604 | 37 | 1949 | 900 | 4325 | 1544 |
| **AgNO_3_** | 5 [3.2] | 404 | 191 | 722 | 335 | 73 | 16 | 87 | 37 | 142 | 35 | 177 | 42 | 81.7 | 33 | 130 | 40 |
|  | 55 [35] | 39789 | 5704 | 41558 | 5668 | 10739 | 2104 | NQ |  | 632 | 129 | 700 | 96 | 4668 | 2919 | 5295 | 3561 |
|  | 125 [80] | 54070 | 21236 | 62335 | 22794 | 46553 | 26985 | 25200 | 4630 | 1091 | 57 | 1019 | 209 | 28859 | 36648 | 16024 | 6464 |
| **AgAc** | 5 [3.25] | 283 | 71 | 990 | 455 | 62 | 14 | 126 | 110 | 142 | 36 | 169 | 21 | 55.4 | 15 | 305 | 232 |
|  | 55 [36] | 38656 | 11000 | 60783 | 10400 | 3500 | 1540 | 4501 | 1420 | 637 | 57 | 805 | 36 | 9471 | 4120 | 16521 | 3610 |
|  | 175 [114] | 96379 | 55200 | 141560 | 93500 | 21373 | 15400 | 46761 | 19800 | 1458 | 33 | 1455 | 114 | 22660 | 7630 | 21872 | 13100 |

|  | **Dose level (mg /kg bw/d)** | **GI tract** | | | | **Ovary** | | **Uterus** | | **Testis** | |
| --- | --- | --- | --- | --- | --- | --- | --- | --- | --- | --- | --- |
|  |  | **M** | | **F** | |  | |  | |  | |
|  |  | Mean | SD | Mean | SD | Mean | SD | Mean | SD | Mean | SD |
| **AgMP** | 36 | 261 | 134 | 310 | 162 | 82 | 32 | <LLOQ |  | 77 | 36 |
|  | 180 | 1004 | 1271 | 3755 | 1853 | 1328 | 917 | 153 | 59 | 213 | 180 |
|  | 1000 | 2620 | 721 | 5124 | 1645 | 2470 | 1273 | 184 | 127 | 296 | 81 |
| **AgNP** | 3.6 | 156 | 43 | 415 | 196 | 116 | 94 | <LLOQ |  | 66 | 27 |
|  | 36 | 3888 | 1799 | 5784 | 726 | 5957 | 2255 | 225 | 133 | 452 | 173 |
|  | 360 | 11466 | 8252 | 9630 | 3721 | 19170 | 6141 | 3865 | 1915 | 1310 | 152 |
| **AgNO_3_** | 5 [3.2] | 1310 | 214 | 2438 | 425 | 1484 | 561 | 145 | 82 | 231 | 103 |
|  | 55 [35] | 5319 | 2412 | 3624 | 1449 | 31357 | 8793 | 8104 | 3075 | 1202 | 246 |
|  | 125 [80] | 10592 | 10669 | 14798 | 7728 | 44025 | 16158 | 22466 | 4327 | 1464 | 260 |
| **AgAc** | 5 [3.25] | 2770 | 1720 | 3797 | 1490 | 2197 | 1100 | 188 | 121 | 167 | 76 |
|  | 55 [36] | 19199 | 7680 | 50736 | 11200 | 24262 | 2610 | 8004 | 3300 | 1508 | 215 |
|  | 175 [114] | 83211 | 5270 | 103814 | 12600 | 39668 | 19800 | 11094 | 2319 | 1531 | 237 |

LLOQ = Lower Limit of Quantification. NQ = Not quantifiable (insufficient sample size).
